# Effects of Short-Term Low- and High-Dose New Zealand Blackcurrant Supplementation on Exercise and Cognitive Performance in Resistance-Trained Adults: A Randomized, Double-Blind, Placebo-Controlled Crossover Study

**DOI:** 10.3390/nu18121929

**Published:** 2026-06-15

**Authors:** Majid S. Koozehchian, Faith M. Bonness, Rafaela Rafajlovska, Shelby N. Horton, Gina Mabrey, Alireza Naderi, Andrew T. Newton

**Affiliations:** 1 Department of Kinesiology, Jacksonville State University, Jacksonville, AL 36265, USA; fbonness@alum.jsu.edu (F.M.B.); rrafajlovska@jsu.edu (R.R.); shorton3@stu.jsu.edu (S.N.H.); gmabrey@jsu.edu (G.M.); atnewton@jsu.edu (A.T.N.); 2 Department of Sport Physiology, Boroujerd Branch, Azad University, Boroujerd 6915136111, Iran; naderi_a@yahoo.com

**Keywords:** New Zealand blackcurrant, anthocyanins, ergogenic aids, sports nutrition, dietary supplements, exercise performance, resistance training, cognitive function

## Abstract

Background: New Zealand blackcurrant (NZBC) is an anthocyanin-rich supplement with reported ergogenic effects in endurance exercise; however, its effects in resistance-trained adults remain largely unexplored. Objective: This study aimed to examine whether seven days of low- or high-dose NZBC supplementation improves resistance exercise performance, anaerobic capacity, and cognitive function in resistance-trained adults. Methods: Twenty resistance-trained adults completed a randomized, double-blind, placebo-controlled crossover trial with four conditions: no-capsule control (CON), placebo (PL), low-dose blackcurrant (LDBC; 250 mg·day^−1^), and high-dose blackcurrant (HDBC; 600 mg·day^−1^), each for seven days. Outcomes included bench press and leg press 1RM, total lifting volume, Tendo-derived bench press power, 30 s Wingate performance, Stroop Color–Word Test scores, readiness, perceived exertion, hemodynamic responses, and adverse events. Results: LDBC and HDBC increased bench press 1RM versus CON and PL, with increases versus CON of +3.33 kg (ES = 0.72; *p* = 0.005) and +2.34 kg (ES = 0.49; *p* = 0.041), respectively. Leg press 1RM was higher in PL, LDBC, and HDBC versus CON, with the largest effects observed for LDBC (+37.2 kg, ES = 1.33; *p* < 0.001) and HDBC (+25.8 kg, ES = 1.11; *p* < 0.001). Leg press total lifting volume was substantially higher with LDBC (+2627 kg, ES = 1.56; *p* < 0.001) and HDBC (+1025 kg, ES = 0.74; *p* = 0.004) versus CON. Bench press volume showed no significant overall treatment effect (*p* > 0.05). For Tendo-derived power, HDBC exceeded PL for peak (+79.5 W; *p* = 0.006) and mean power (+46.2 W; *p* = 0.026). Wingate outcomes did not differ across conditions (all *p* > 0.05). LDBC exceeded PL on Stroop Color, Color–Word, and total scores (all *p* < 0.05); HDBC exceeded PL on Color–Word only. Hemodynamic responses and adverse events were comparable across all conditions. Conclusions: Short-term NZBC supplementation improved selected resistance-exercise and cognitive outcomes, with the strongest evidence observed for outcomes that exceeded both CON and PL. The PL response relative to CON suggests that non-specific capsule, expectancy, repeated testing, or period effects may have contributed to some of the lower-body improvements; therefore, placebo-controlled contrasts should be emphasized when interpreting NZBC-specific efficacy. Wingate performance was unaffected, and both doses were well tolerated over the short-term supplementation period.

## 1. Introduction

Polyphenol-rich supplements have attracted considerable research interest because anthocyanins and related bioactives may influence exercise-relevant vascular and metabolic processes. In the vascular system, anthocyanins and their metabolites may support endothelial function by increasing nitric oxide bioavailability, modulating endothelial nitric oxide synthase activity, reducing nitric oxide scavenging by reactive oxygen species, and influencing vasoregulatory factors such as endothelin-1 [1,2,3]. These actions may improve peripheral blood flow and local perfusion, supporting oxygen delivery, metabolite clearance, and fatigue resistance during high-intensity or repeated-effort exercise. New Zealand blackcurrant (NZBC) has emerged as one of the more studied sources in this category. Its extract has been shown to improve high-intensity intermittent running, cycling time-trial performance, repeated cycling efforts, and fat oxidation during exercise [4,5,6]. An acute dose also improved 5 km running performance in trained male runners, though without corresponding changes in physiological or metabolic indices [7]. A systematic review and meta-analysis reported generally positive but modest effects of NZBC supplementation on sport performance and related biomarkers, with outcomes appearing to depend on the exercise task, dosing strategy, and participant population [1].

NZBC extract draws most of its bioactive potential from four anthocyanin glycosides, including two delphinidin derivatives, delphinidin-3-rutinoside and delphinidin-3-glucoside, and two cyanidin derivatives, cyanidin-3-rutinoside and cyanidin-3-glucoside, which together represent the bulk of its reported anthocyanin content [2]. These compounds are thought to act through convergent mechanisms, including modulation of vascular responsiveness, oxidative stress, substrate use, and fatigue tolerance [1,2,5]. Each pathway is plausibly relevant during high-intensity or repeated-effort exercise, where perfusion, fatigue resistance, and metabolic efficiency collectively determine performance.

Critically, the picture from endurance and intermittent exercise does not translate neatly to resistance settings. Individual responses to repeated NZBC dosing during high-intensity intermittent treadmill running have been highly variable [2]. A recent trial in trained cyclists found no effect of either acute or 7-day blackcurrant extract on cycling performance or physiological responses [8], and a separate investigation reported no change in force output, muscle activity, cardiovascular function, or fatigue indices during sustained low-intensity isometric contractions following 4 or 7 days of NZBC intake in men [9]. These null findings matter because they expose the limits of extrapolating from one exercise context to another. Resistance exercise places demands on neuromuscular force production, load management across multiple sets, movement technique, and the ability to sustain high-force output as local fatigue accumulates, a profile that differs substantially from cycling, treadmill running, or isometric protocols. Whether maximal strength, total lifting volume, or barbell power responds to NZBC supplementation is therefore a genuinely open question.

Addressing it requires instruments suited to the resistance exercise context. Linear position transducers, such as the Tendo unit, can continuously capture barbell displacement-time data during lifting, yielding mean and peak power estimates that reflect neuromuscular output across repetitions [10]. For anaerobic capacity, the 30 s Wingate test provides well-validated indices of peak and mean power and total anaerobic work, with established methodological standards [11,12,13]. Using both alongside traditional 1RM and volume measures allows for a more complete characterization of whether NZBC supplementation influences resistance performance specifically, or high-intensity exercise more broadly.

Cognitive function is a less-examined but practically relevant dimension of supplement research in athletic populations. Training and competition require attention, inhibitory control, and rapid information processing under physical and psychological stress. The Stroop Color–Word Test is a well-established measure of executive function and cognitive control [14,15,16], but it has rarely been evaluated alongside physical performance outcomes in NZBC supplementation trials. Including it in the present study allowed us to examine whether NZBC influenced cognitive performance under the same experimental conditions as the physical endpoints. This rationale is supported by evidence that blackcurrant-derived bioactives can inhibit monoamine oxidase-B, which may help preserve monoamine neurotransmitter availability and provide a plausible pathway for effects on attention, inhibitory control, and cognitive performance [17].

This study therefore investigated the effects of seven days of NZBC supplementation at two doses on resistance exercise performance and cognitive function in resistance-trained adults, using four conditions: a no-capsule control (CON), placebo (PL), low-dose blackcurrant (LDBC; 250 mg·day^−1^), and high-dose blackcurrant (HDBC; 600 mg·day^−1^). Primary outcomes were maximal strength, total lifting volume, Tendo-derived bench press power, Wingate anaerobic performance, and Stroop Color–Word Test performance. Secondary outcomes included readiness, perceived exertion, hemodynamic responses, and adverse events. We hypothesized that NZBC supplementation would improve selected resistance-exercise, anaerobic, and cognitive outcomes relative to CON and PL, with dose-specific response patterns, and without adverse hemodynamic or tolerability effects.

## 2. Materials and Methods

### 2.1. Study Design, Oversight, and Reporting

**Figure 1:**
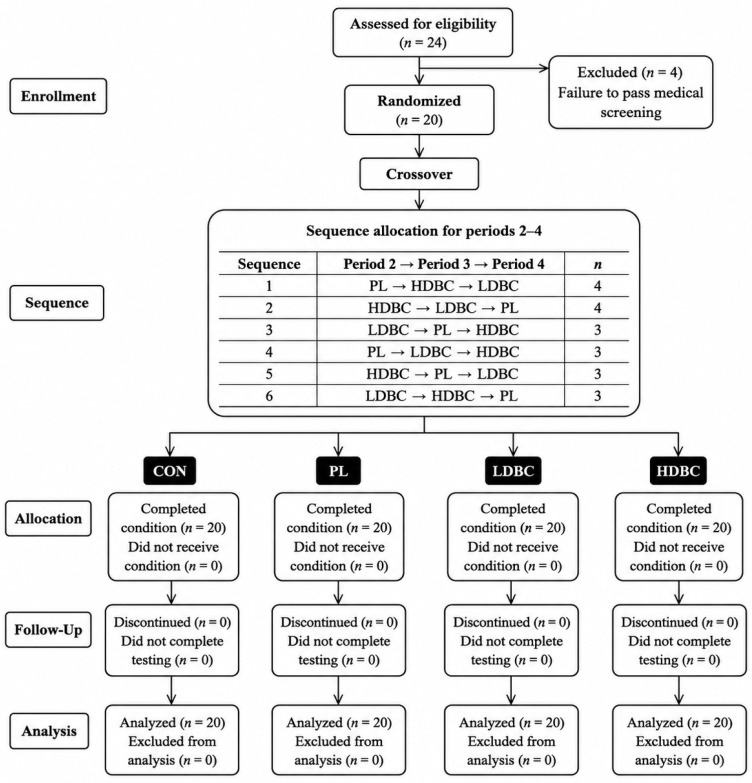
CONSORT 2025 flow diagram for the randomized crossover trial. Twenty-four participants were assessed for eligibility; four were excluded during medical screening, and 20 were randomized and analyzed. CON was completed first for all participants, followed by the three blinded capsule conditions in periods 2–4 according to a near-balanced Williams-type sequence allocation. All 20 participants completed each condition and were included in the analysis.

**Figure 2:**
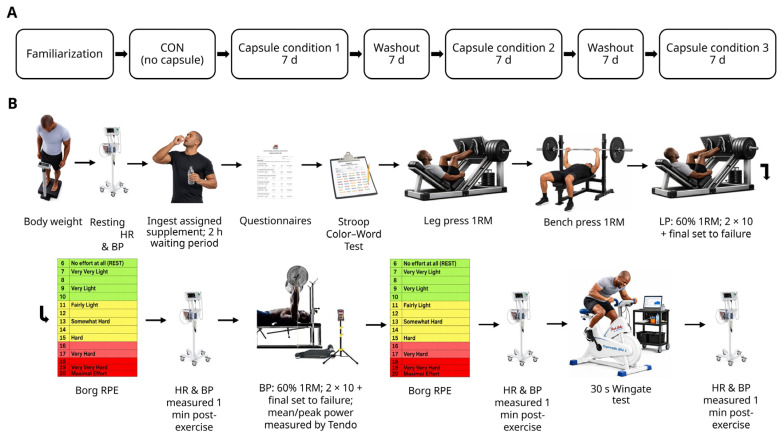
Study workflow and testing sequence. (**A**) Participants completed a familiarization session, followed by the no-capsule control condition (CON) and three randomized 7-day capsule conditions (PL, LDBC, and HDBC), each separated by a 7-day washout. (**B**) Capsule-visit testing sequence: body mass, resting HR and BP → capsule ingestion → 2 h waiting period with questionnaires → Stroop Color–Word Test → leg press 1RM → bench press 1RM → leg press at 60% 1RM (2 × 10 + final set to failure) → bench press at 60% 1RM (2 × 10 + final set to failure; mean/peak power measured by Tendo) → 30 s Wingate test. Borg RPE was recorded immediately after the final sets of the leg press and bench press. HR and BP were recorded 1 min after the final leg press set, final bench press set, and Wingate test. During CON, no capsule was administered, and the 2 h waiting period was omitted.

In this investigation, participants completed a randomized, double-blind, placebo-controlled crossover protocol in the Exercise Physiology and Nutrition Laboratory (EPNL) of the Department of Kinesiology at Jacksonville State University. This study was conducted in accordance with the Declaration of Helsinki and was approved by the institutional review board (IRB #12132022-01). The trial was retrospectively registered at ClinicalTrials.gov (Identifier: NCT07619976) on 1 June 2026. This trial is reported in accordance with CONSORT guidelines, including extensions for randomized crossover trials [18]. Participants completed four conditions: CON, PL, LDBC, and HDBC. CON was conducted as the first post-familiarization visit, and the three capsule conditions were conducted afterward in a randomized order. Each capsule condition lasted seven consecutive days, with laboratory testing on day 7. During each 7-day loading period, participants took one capsule daily according to their training schedule: 2 h before exercise on training days and at noon on non-training days; on laboratory testing days, the final capsule was administered on-site after initial check-in procedures and 2 h before cognitive and performance testing. Figure 1 illustrates the screening, randomization, allocation, follow-up, and analysis steps. Figure 2 illustrates the four-period study workflow and the sequence of assessments administered at each visit. No capsule was administered during CON. No separate public trial protocol or statistical analysis plan was available before or during this study; the study procedures and statistical analyses are described in Section 2.1, Section 2.2, Section 2.3, Section 2.4, Section 2.5, Section 2.6, Section 2.7, Section 2.8 and Section 2.9. No patient or public involvement was used in the design, conduct, or reporting of the trial. No important changes to the intervention procedures, outcome assessments, or planned statistical approach were made after trial commencement. Trial registration URL: https://clinicaltrials.gov/study/NCT07619976 (accessed on 12 May 2026). This trial used an exploratory framework.

### 2.2. Participants and Eligibility

Participants were recruited through campus advertisements and email outreach. Inclusion criteria were age 18–40 years; at least 2 years of regular resistance training that included the bench press and either the leg press or squat; and a body mass index of 18.5–24.9 kg·m^−2^, consistent with the WHO normal-weight BMI classification [19]. Exclusion criteria included any diagnosed metabolic, cardiovascular, including cardiac arrhythmias, or thyroid disorder; current use of prescription medications, over-the-counter medications, or stimulant-containing products with potential cardiovascular or neurocognitive effects; tobacco use, vaping, or smokeless nicotine use; self-reported blackcurrant sensitivity; habitual alcohol intake > 12 standard drinks per week; and any recent musculoskeletal injury likely to impair testing. During familiarization, participants were excluded if their seated resting heart rate (HR) exceeded 100 bpm, seated blood pressure (BP) was ≥140/90 mmHg after repeat confirmation [20], or DXA-derived body fat percentage exceeded the predefined threshold of 25% for males or 35% for females. A registered nurse collected medical history and performed a brief physical examination during familiarization to confirm eligibility, as described in Section 2.3.

BMI was used as an initial health-screening criterion rather than as the sole indicator of adiposity, because BMI may misclassify resistance-trained individuals with greater lean mass. Therefore, DXA was also used to assess body composition during screening. These DXA-derived body fat thresholds were selected a priori using guidance from the American College of Sports Medicine and Gallagher et al. [21,22]. They were applied strictly as upper-bound health-screening criteria, not as criteria for athletic leanness, to exclude excessive adiposity that could potentially confound health screening, supplement distribution, or performance outcomes in this resistance-trained cohort.

Menstrual-cycle phase was not prospectively standardized across testing sessions, and hormonal contraceptive use was not used as an inclusion or exclusion criterion.

Recruitment was not stratified to obtain equal numbers of males and females. The final analyzed sample included 7 males and 13 females. Participant recruitment and study visits occurred from January 2023 to May 2023. The trial ended after completion of the planned data collection, and no early stopping occurred.

### 2.3. Screening, Familiarization, and Standardization

After a phone pre-screen, eligible participants completed a familiarization visit during which they provided written informed consent, completed medical and training-history questionnaires, and were briefed on all study procedures. At this visit, stature, body mass, body composition, and resting hemodynamics, including HR, systolic BP (SBP), and diastolic BP (DBP), were assessed. Height and body mass were measured using a digital physician scale with an integrated stadiometer (Health o meter Professional 500KL; Pelstar LLC, McCook, IL, USA), with body mass recorded to the nearest 0.1 kg and stature recorded to the nearest 0.01 m. Total body composition was assessed using dual-energy X-ray absorptiometry (DXA; Hologic Horizon A, Hologic Inc., Marlborough, MA, USA). The same technician performed all scans in accordance with the manufacturer’s instructions and laboratory SOPs. Afterward, participants sat quietly for ≥10 min, and resting HR, SBP, and DBP were measured using an automated oscillometric device (Connex ProBP 3400; Welch Allyn, Skaneateles Falls, NY, USA), consistent with current BP measurement recommendations [20]. Subsequent visits were time-matched within participants to limit circadian effects, and ambient temperature and relative humidity were monitored and logged at each session; laboratory conditions were maintained at 21–22 °C and 50–60% relative humidity.

During familiarization, participants completed leg-press and bench-press 1RM assessments using consistent warm-up procedures and established resistance-training assessment principles [23]. They then practiced the 30 s Wingate Anaerobic Test to minimize learning effects before the experimental trials and to improve protocol consistency across test days [11,12,13]. Participants received uniform instructions for completing the readiness-to-perform survey, side-effect questionnaire, and Stroop Color–Word Test. To promote consistency across visits, participants were instructed to follow a regular sleep-wake cycle and comply with the pre-visit restrictions described in Section 2.6.

### 2.4. Randomization, Allocation Concealment, and Blinding

All participants completed CON in session 1. The three capsule conditions were assigned to periods 2–4 using a balanced 3 × 3 Williams design to minimize first-order carryover among PL, LDBC, and HDBC. Randomization was computer-generated by an investigator independent of data collection, and capsule-condition sequences were concealed in sequentially numbered, opaque, sealed envelopes prepared by an impartial coordinator. Capsules for PL, LDBC, and HDBC were identical in appearance and mass and were dispensed in coded packaging; therefore, both participants and outcome assessors were blinded to capsule condition. The CON visit could not be participant-blinded because no capsule was administered; to limit expectation effects, consistent instructions and procedures were used across all visits. Personnel involved in enrollment and outcome assessment did not have access to the concealed capsule-condition sequence before assignment.

### 2.5. Interventions and Dosing Regimen

Four conditions were completed: CON (no capsule), PL (maltodextrin 600 mg·day^−1^), LDBC (NZBC 250 mg·day^−1^ + maltodextrin 350 mg·day^−1^), and HDBC (NZBC 600 mg·day^−1^). The NZBC supplement was prepared from CurraNZ^®^ New Zealand Blackcurrant Extract (Health Currancy Ltd., Camberley, Surrey, UK), which, according to the manufacturer’s product information, is a 35% anthocyanin extract. Maltodextrin was obtained from BulkSupplements.com (Henderson, NV, USA) and was used as the placebo material and capsule filler.

The original CurraNZ capsules were opened, and the extract was reweighed and re-encapsulated to provide 250 mg NZBC extract for LDBC and 600 mg NZBC extract for HDBC. Maltodextrin was used to standardize capsule mass across the blinded capsule conditions; therefore, LDBC contained 250 mg NZBC plus 350 mg maltodextrin to match the 600 mg total capsule mass used in PL and HDBC.

The LDBC and HDBC doses were selected to represent low- and high-dose NZBC conditions within the range commonly examined in previous short-term athlete and physically active participant studies [4,5,6], and are consistent with systematic evidence on NZBC supplementation and sport performance [1]. In PL, LDBC, and HDBC, participants took one capsule daily for 7 consecutive days. Capsules were taken 2 h before workouts on training days and at noon on non-training days; on the laboratory test day, the final capsule was administered on-site 2 h before testing, consistent with prior short-term and acute NZBC supplementation protocols [1,2,6].

Based on the manufacturer/product specification of approximately 35% anthocyanins, the LDBC capsule with 250 mg NZBC provided approximately 87.5 mg anthocyanins·day^−1^, whereas the HDBC capsule with 600 mg NZBC provided approximately 210 mg anthocyanins·day^−1^. Based on midpoint composition values for the major anthocyanins, including delphinidin-3-rutinoside (42.5%), delphinidin-3-glucoside (12.5%), cya-nidin-3-rutinoside (37.5%), and cyanidin-3-glucoside (6.5%), the estimated daily intakes for LDBC were approximately 37.2, 10.9, 32.8, and 5.7 mg, respectively, whereas the estimated daily intakes for HDBC were approximately 89.3, 26.3, 78.8, and 13.7 mg, respectively [2].

### 2.6. Pre-Visit Restrictions, Adherence, and Co-Interventions

For each experimental testing session, participants reported to the laboratory during morning hours, typically between 6:00 a.m. and 12:00 p.m., after an overnight fast of at least 8 h. Testing visits were time-matched within participants to minimize circadian variation.

Standardization began two weeks before the first test session, when participants discontinued dietary and sports supplements with potential ergogenic or physiological effects, including creatine, caffeine-containing pre-workouts, nitrate products, polyphenol supplements, herbal products, and similar performance aids. Protein powders were discontinued when used as supplemental products, while habitual dietary protein intake was monitored through dietary records. In the 72 h before each laboratory visit, they abstained from structured exercise, caffeine, alcohol, dietary nitrate, polyphenol-rich foods, and antibacterial mouthwash. Outside the 72 h pre-visit restriction period, participants were instructed to maintain their habitual training routines and avoid initiating any new exercise program during this study; however, physical activity outside this restriction period was not quantified using standardized physical-activity questionnaires or metabolic equivalent (MET)-based estimates. Staff checked compliance on arrival using a brief checklist; any non-compliant session was rescheduled. Capsule adherence was tracked through daily dosing logs, and the final dose for each capsule condition was administered on-site under direct staff observation on the test day.

### 2.7. Outcome Measures and Instruments

#### 2.7.1. Performance Testing

Laboratory environmental conditions remained within the predefined ranges throughout testing. Upon arrival, participants completed the visit-specific screening procedures and body mass assessment before performance testing. Strength testing included the bench press and leg press. At each visit, a 1RM test was performed for each lift to assess day-to-day strength performance; however, all subsequent work sets were completed at 60% of the familiarization 1RM, which was held constant across all sessions. Participants then completed three work sets at the prescribed load with 2 min of inter-set rest: sets 1 and 2 were fixed at 10 repetitions, whereas set 3 was continued to volitional failure [23]. Training volume was calculated as Σ(load × repetitions) across the three sets. During the bench press, a linear position transducer (Tendo Unit, Tendo Sport Machines, Trenčín, Slovakia) recorded bar displacement over time. Software-derived mean and peak power values for each repetition were averaged across repetitions in set 3 to generate set-level outcomes [10]. All strength evaluations were conducted using the same procedures with a flat bench and a hip/leg sled (Hammer Strength, Schiller Park, IL, USA) [24].

Anaerobic capacity was assessed using a 30 s Wingate test on a friction-braked cycle ergometer (Monark 894E Peak Bike, Monark, Vansbro, Sweden) with a relative braking force of 0.075 kg·kg^−1^ body mass [11]. Saddle and handlebar positions were recorded during the first visit and replicated at each subsequent visit. Toe clips/straps were used to minimize foot slippage. The warm-up comprised easy pedaling interspersed with five 5 ss accelerations to approximately 70 rpm, each separated by roughly 1 min of light cycling. After a standardized countdown, participants completed a seated all-out 30 s sprint; the braking load was applied once cadence reached approximately 90–100 rpm, typically within the first 3 s, to limit start-up inertia effects [12,13]. Verbal encouragement and time cues were provided uniformly every 5–10 s, followed by a 2–3 min easy cycling cool-down. Power output was continuously recorded and processed using the device software. Peak power was the highest output recorded over any single second of the sprint; mean power was the average across all 30 s; and total work was the cumulative mechanical output for the full effort. If foot slippage or an early termination compromised a trial, it was discarded and repeated once the participant had recovered adequately.

#### 2.7.2. Cognitive and Subjective Assessments

Cognitive function was assessed using the paper-and-pencil Stroop Color–Word Test [14,15,16]. Testing was conducted in a quiet, seated setting, with manual-based instructions read verbatim. The test consisted of three consecutive 45 ss sections administered in the standard order: (i) Word, in which participants read color names printed in black ink; (ii) Color, in which participants named the ink color of “XXXX” strings; and (iii) Color–Word, in which participants named the ink color of incongruent color words, such as the word “RED” printed in blue ink. Responses were provided aloud and tallied in real time. For each section, the score was the number of correct responses completed within 45 s. Errors were not credited; immediate self-corrections were permitted and scored according to the test manual. The primary cognitive outcome was the total number of correct responses across all three sections, while section-specific scores were analyzed as secondary outcomes. Before testing, participants completed a six-item readiness-to-perform questionnaire using anchored 1–5 response scales to assess sleep quality, motivation to exercise, optimism about performance, vigor/energy, appetite, and muscle soreness, consistent with subjective athlete-monitoring approaches [25].

#### 2.7.3. Hemodynamic and Perceptual Responses; Tolerability

Resting HR and BP were obtained at check-in after ≥10 min of seated, quiet rest, following the procedures described in Section 2.3 and current recommendations for BP assessment [20]. Post-exercise HR and BP were recorded at three predefined 1 min recovery time points: after the third set of the bench press, after the third set of the leg press, and after completion of the Wingate test. All measurements were obtained using the same automated device, arm, seated posture, limb position, and cuff-selection procedures described in Section 2.3, with participants seated, feet flat, back supported, and the arm positioned at heart level [20]. If motion or artifact was detected, the measurement was repeated immediately according to device guidance. Rating of Perceived Exertion (RPE; Borg 6–20 scale) was recorded immediately after the final sets of the bench press and leg press [26]. Tolerability was evaluated during the capsule conditions using a brief side-effects questionnaire, with responses documented on case-report forms. This questionnaire was not administered during familiarization or CON because no supplement was provided.

### 2.8. Dietary Monitoring and Standardization

Dietary intake was recorded using MyFitnessPal (Version 22.24.2) across four assessment windows, each covering three consecutive days (two weekdays and one weekend day where possible), giving up to 12 recording days in total. Three-day records combining weekday and weekend days are a well-established approach in nutrition epidemiology [27]. For each entry, participants logged all foods, beverages, and supplements consumed, noting the brand or manufacturer, preparation method, and portion size in household measures or grams; barcode scanning and nutrition labels were used where available, and custom foods and recipes were saved for items that recurred across the study period. Before the first window opened, a brief training session covered portion-size estimation and the core app functions relevant to this study. At the close of each window, a staff member reviewed the logs with the participant to resolve missing entries, ambiguous portion sizes, and restaurant meals. MyFitnessPal has been validated for nutrient estimation, and the broader literature on smartphone-based dietary records supports this approach for monitoring intake in research settings, with the caveat that self-report methods carry inherent limitations [28,29].

For analysis, daily energy intake and macronutrient intake were extracted from MyFitnessPal and averaged across the three recording days for each condition. Energy intake was expressed as kcal/day, and protein, carbohydrate, and fat were expressed as g/day. Condition-specific averages were compared across CON, PL, LDBC, and HDBC as described in Section 2.9.

### 2.9. Statistical Analysis

All analyses were conducted using a four-condition, repeated-measures crossover design comprising CON, PL, LDBC, and HDBC. Each outcome was analyzed using a repeated-measures general linear model with treatment (TRT; 4 levels) as the within-subject factor. The primary model tested the main effect of treatment. Residual normality was assessed using the Shapiro–Wilk test and diagnostic plots. Sphericity was evaluated using Mauchly’s test, and Greenhouse–Geisser corrections were applied when sphericity was violated. When the overall treatment effect was significant, pairwise comparisons were performed using estimated marginal means with the least significant difference procedure. No additional familywise multiple-comparison correction, such as Bonferroni, Holm, or false discovery rate adjustment, was applied across pairwise contrasts or outcomes. For outcomes with a non-significant overall treatment effect, pairwise comparisons were considered exploratory and interpreted cautiously alongside effect sizes and confidence intervals.

No formal a priori sample size calculation was performed because this was an exploratory, feasibility-constrained crossover trial; the final sample size was based on recruitment feasibility, laboratory constraints, and comparable short-term NZBC supplementation studies. No interim analyses or formal stopping guidelines were planned or performed.

Because this study was not sufficiently powered to evaluate sex-specific treatment responses, sex-based inferential analyses, including testing for treatment-by-sex interactions and sex-stratified interpretation of treatment responses, were removed from the primary analysis.

Baseline characteristics are reported descriptively by sex and for the total sample. Dietary intake data are reported for the overall sample. Treatment outcomes are reported as model-based estimated marginal means ± SE for the overall sample. Repeated-measures ANOVA results are presented as *p*-values for the main effect of treatment. When the overall treatment effect was significant, pairwise differences among conditions were identified using superscript letters in tables and further illustrated in figures using model-based mean differences with 95% confidence intervals relative to CON. For pairwise comparisons, effect sizes were calculated as Cohen’s dz using the formula t/√*n* and interpreted as trivial (<0.20), small (0.20–0.49), moderate (0.50–0.79), or large (≥0.80). Because missing observations were minimal, analyses used listwise deletion with no imputation. All statistical analyses were conducted using IBM SPSS Statistics, version 31 (IBM Corp., Armonk, NY, USA).

## 3. Results

### 3.1. Participants

Analyses included all 20 participants who completed all four study periods. Baseline characteristics are presented in Table 1.

### 3.2. Dietary Intake

Dietary intake is summarized in Table 2. No significant treatment effects were observed for energy intake or macronutrient intake (all *p* > 0.05), indicating that self-reported dietary intake was generally consistent across CON, PL, LDBC, and HDBC.

### 3.3. Primary Outcomes

#### 3.3.1. Exercise Performance

Treatment effects are reported for the overall sample. Sex-specific treatment-response analyses were not conducted because this study was not powered to evaluate them.

**Figure 3:**
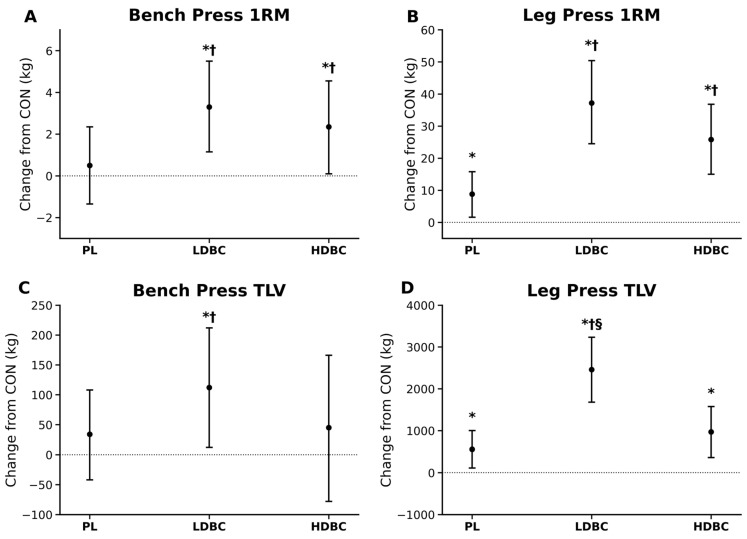
Changes in performance relative to the CON session. (**A**) Bench press 1RM; (**B**) Leg press 1RM; (**C**) Bench press total lifting volume; (**D**) Leg press total lifting volume. Points show adjusted means with 95% confidence intervals for each treatment. The dotted horizontal line indicates no difference from CON. Significance markers denote pairwise differences: * vs. CON; † vs. PL; § vs. HDBC (*p* < 0.05).

Bench press 1RM (kg): A significant treatment effect was observed for bench press 1RM (TRT: *p* = 0.003). LDBC and HDBC were both higher than CON (+3.33 kg; 95% CI 1.16 to 5.50; *p* = 0.005; +4.8%; ES = 0.72, moderate; and +2.34 kg; 95% CI 0.11 to 4.57; *p* = 0.041; +3.3%; ES = 0.49, small-to-moderate, respectively) and higher than PL (+2.84 kg; 95% CI 1.01 to 4.68; *p* = 0.004; +4.0%; ES = 0.73, moderate; and +1.86 kg; 95% CI 0.61 to 3.10; *p* = 0.006; +2.6%; ES = 0.70, moderate, respectively). PL did not differ from CON (ES = 0.12), and LDBC did not differ from HDBC (ES = 0.31) (Table 3; Figure 3A).

Leg press 1RM (kg): A significant treatment effect was observed for leg press 1RM (TRT: *p* < 0.001). PL, LDBC, and HDBC were each higher than CON, with the largest effects observed for LDBC (+37.2 kg; 95% CI 24.1 to 50.3; *p* < 0.001; +13.0%; ES = 1.33, large) and HDBC (+25.8 kg; 95% CI 14.9 to 36.7; *p* < 0.001; +9.0%; ES = 1.11, large), compared with the smaller PL response (+8.81 kg; 95% CI 1.67 to 15.9; *p* = 0.018; +3.1%; ES = 0.58, moderate). Both blackcurrant conditions also exceeded PL (LDBC: +28.4 kg; *p* = 0.001; +9.6%; ES = 0.85, large; HDBC: +17.1 kg; *p* = 0.003; +5.8%; ES = 0.75, moderate), whereas LDBC and HDBC did not differ significantly (ES = 0.41) (Table 3; Figure 3B).

Bench press total lifting volume (kg): The overall treatment effect for bench press total lifting volume was not significant after Greenhouse–Geisser correction (TRT: *p* = 0.102). Therefore, pairwise findings were interpreted as exploratory. LDBC showed higher values than CON (+111.8 kg; 95% CI 12.2 to 211.5; *p* = 0.030; +7.8%; ES = 0.53, moderate) and PL (+79.3 kg; 95% CI 20.3 to 138.2; *p* = 0.011; +5.4%; ES = 0.63, moderate), whereas PL versus CON (ES = 0.20), HDBC versus CON (ES = 0.17), HDBC versus PL (ES = 0.06), and LDBC versus HDBC (ES = 0.42) were not significant (Table 3; Figure 3C).

Leg press total lifting volume (kg): Total leg press lifting volume differed significantly across treatments (TRT: *p* < 0.001). LDBC showed the largest increase relative to CON (+2627.0 kg; 95% CI 1835.5 to 3418.5; *p* < 0.001; +38.1%; ES = 1.56, large), followed by HDBC (+1024.6 kg; 95% CI 374.9 to 1674.2; *p* = 0.004; +14.9%; ES = 0.74, moderate) and PL (+520.1 kg; 95% CI 43.1 to 997.1; *p* = 0.034; +7.5%; ES = 0.51, moderate). LDBC also exceeded PL (+2106.9 kg; 95% CI 1436.3 to 2777.6; *p* < 0.001; +28.4%; ES = 1.48, large) and HDBC (+1602.4 kg; 95% CI 1078.6 to 2126.3; *p* < 0.001; +20.2%; ES = 1.44, large), whereas HDBC did not differ significantly from PL (ES = 0.40) (Table 3; Figure 3D).

**Figure 4:**
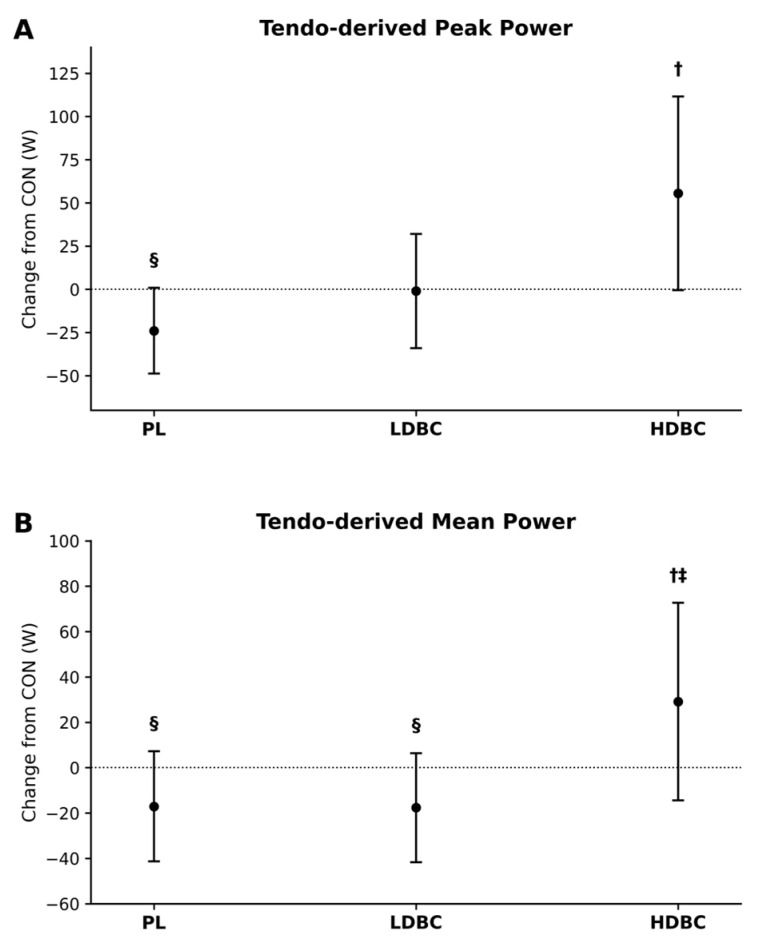
Tendo-derived power changes relative to the CON session. (**A**) Peak power; (**B**) mean power. Data points represent adjusted means with 95% confidence intervals for each treatment condition. The dotted horizontal line indicates no change relative to CON. Significance markers indicate pairwise differences at *p* < 0.05: † vs. PL; ‡ vs. LDBC; § vs. HDBC. Power values are reported in watts.

Tendo peak power (W): A significant treatment effect was observed for Tendo peak power (TRT: *p* = 0.017). The clearest difference was between HDBC and PL, with HDBC producing higher peak power (+79.5 W; 95% CI 26.4 to 132.6; *p* = 0.006; +25.8%; ES = 0.70, moderate). HDBC also showed a positive, but non-significant, difference compared with CON (+55.5 W; 95% CI −0.51 to 111.6; *p* = 0.052; +16.7%; ES = 0.47, small-to-moderate). PL versus CON (ES = −0.45), LDBC versus CON (ES = −0.01), LDBC versus PL (ES = 0.31), and LDBC versus HDBC (ES = −0.43) were not significant (Table 3; Figure 4A).

Tendo mean power (W): A significant treatment effect was also observed for Tendo mean power (TRT: *p* = 0.038). HDBC was higher than PL (+46.2 W; 95% CI 6.23 to 86.1; *p* = 0.026; +21.7%; ES = 0.54, moderate) and higher than LDBC (+46.9 W; 95% CI 5.07 to 88.6; *p* = 0.030; +22.1%; ES = 0.53, moderate). Differences versus CON were not significant for PL (ES = −0.33), LDBC (ES = −0.35), or HDBC (ES = 0.31), and LDBC did not differ from PL (ES = −0.04) (Table 3; Figure 4B).

Wingate anaerobic performance: No significant treatment effects were observed for Wingate peak power (TRT: *p* = 0.102), mean power (TRT: *p* = 0.611), or total work (TRT: *p* = 0.784). Pairwise comparisons versus CON were also non-significant for all Wingate outcomes (*p* > 0.05), indicating no significant treatment effect on anaerobic cycling performance in this protocol. (Table 4).

#### 3.3.2. Effects on Cognitive Function

Treatment effects are reported for the overall sample. Sex-specific cognitive treatment-response analyses were not conducted because this study was not powered to evaluate them.

**Figure 5:**
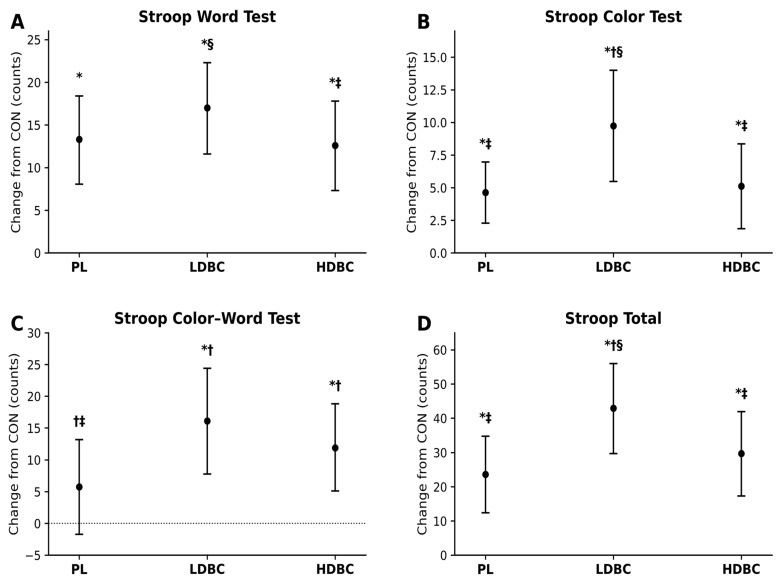
Stroop Color–Word Test results compared with the CON session: Word (**A**), Color (**B**), Color–Word (**C**), and Total (**D**). Points show adjusted means with 95% confidence intervals for each treatment. The dotted horizontal line indicates no difference from CON. Significance markers denote pairwise differences: * vs. CON; † vs. PL; ‡ vs. LDBC; § vs. HDBC (*p* < 0.05).

Word: A significant treatment effect was detected for the Word section of the Stroop Color–Word Test (TRT: *p* < 0.001). PL, LDBC, and HDBC were all higher than CON (+13.3 to +17.0 counts; *p* < 0.001; +12.5% to +16.0%; ES = 1.13 to 1.49), with the largest increase observed for LDBC. Among capsule conditions, LDBC was higher than HDBC (+4.44 counts; 95% CI 0.49 to 8.39; *p* = 0.030; +3.7%; ES = 0.53), whereas PL did not differ from LDBC or HDBC (Figure 5A; Table 5).

Color: A significant treatment effect was detected for the Color section (TRT: *p* < 0.001). PL, LDBC, and HDBC were higher than CON (+4.63 to +9.74 counts; *p* < 0.05; +5.8% to +12.2%; ES = 0.74 to 1.07). LDBC also exceeded PL (+5.11 counts; 95% CI 0.99 to 9.23; *p* = 0.018; +6.1%; ES = 0.58) and HDBC (+4.63 counts; 95% CI 0.71 to 8.54; *p* = 0.023; +5.5%; ES = 0.55), whereas PL and HDBC did not differ (Figure 5B; Table 5).

Color–Word: A significant treatment effect was observed for the Color–Word section (TRT: *p* = 0.002). LDBC and HDBC were higher than CON (+16.1 and +11.9 counts; *p* < 0.01; +16.3% and +12.1%; ES = 0.91 and 0.81, respectively), whereas PL did not differ from CON (*p* = 0.123; ES = 0.36). Compared with PL, both LDBC (+10.3 counts; 95% CI 4.20 to 16.4; *p* = 0.002; +9.9%; ES = 0.79) and HDBC (+6.23 counts; 95% CI 1.20 to 11.2; *p* = 0.018; +6.0%; ES = 0.58) were higher, while LDBC and HDBC did not differ from each other (Figure 5C; Table 5).

Total Stroop score: A significant treatment effect was detected for total Stroop performance (TRT: *p* < 0.001). PL, LDBC, and HDBC were all higher than CON (+23.6 to +42.9 counts; *p* < 0.001; +8.3% to +15.1%; ES = 0.99 to 1.53). LDBC produced the largest improvement and was higher than both PL (+19.2 counts; 95% CI 8.18 to 30.2; *p* = 0.002; +6.2%; ES = 0.82) and HDBC (+13.2 counts; 95% CI 1.49 to 24.8; *p* = 0.029; +4.2%; ES = 0.53), whereas PL did not differ from HDBC (Figure 5D; Table 5).

### 3.4. Secondary Outcomes

#### 3.4.1. Perceptual Responses (VAS Readiness and RPE)

Visual Analog Scale for Readiness to Perform: Perceived readiness was assessed using VAS items measuring sleep quality, motivation to exercise, anticipated performance, energy/vigor, appetite, and muscle soreness. Across all readiness-related items, the primary ANOVA showed no significant treatment effect. Pairwise comparisons were also non-significant, indicating that readiness scores were comparable across CON, PL, LDBC, and HDBC conditions (*p* ≥ 0.05).

Rating of perceived exertion: Post-bench press RPE differed significantly across treatments (TRT: *p* = 0.044). Adjusted means (±SE) were CON = 15.5 ± 0.54, PL = 16.0 ± 0.51, LDBC = 16.7 ± 0.51, and HDBC = 16.5 ± 0.55. Pairwise comparisons showed that LDBC (+1.20; 95% CI 0.20 to 2.20; *p* = 0.021) and HDBC (+0.93; 95% CI 0.04 to 1.82; *p* = 0.042) were higher than CON, whereas PL did not differ from CON (*p* = 0.150). No significant differences were observed among PL, LDBC, and HDBC (*p* > 0.05).

Post-leg press RPE also differed significantly across treatments (TRT: *p* < 0.001). Adjusted means (±SE) were CON = 14.7 ± 0.48, PL = 15.2 ± 0.69, LDBC = 16.8 ± 0.58, and HDBC = 16.3 ± 0.54. Pairwise comparisons showed that LDBC (+2.08; 95% CI 1.19 to 2.98; *p* < 0.001) and HDBC (+1.54; 95% CI 0.60 to 2.47; *p* = 0.003) were higher than CON, whereas PL did not differ from CON (*p* = 0.310). LDBC was also higher than PL (+1.58; 95% CI 0.48 to 2.68; *p* = 0.007), while HDBC did not differ from PL (*p* = 0.069) or LDBC (*p* = 0.151).

#### 3.4.2. Hemodynamic Responses: Resting and 1 Min Post-Exercise HR and BP

HR and BP were generally similar across treatment conditions. No significant treatment effects were observed for resting HR or resting BP. Similarly, 1 min post-exercise HR and BP did not differ across conditions following the bench press, leg press, or Wingate tests. Collectively, these findings indicate that CON, PL, LDBC, and HDBC produced comparable hemodynamic responses at rest and immediately after exercise.

#### 3.4.3. Adverse Events

Participants were monitored with a side-effects questionnaire assessing a broad range of physical and psychological symptoms. Across treatment conditions, neither the overall side-effects index nor individual symptom scores differed significantly (*p* > 0.05). Reported symptoms were generally absent or mild, and no serious adverse events occurred during this study.

## 4. Discussion

This study examined the effects of short-term NZBC supplementation at low and high doses on exercise performance, cognitive outcomes, perceptual responses, hemodynamic responses, and tolerability in resistance-trained adults. To our knowledge, this is among the first studies to evaluate whether low- and high-dose NZBC differentially affect maximal strength, repeated-set resistance performance, Tendo-derived barbell power, anaerobic cycling performance, and Stroop-based cognitive function in this population. Overall, NZBC improved selected resistance exercise and cognitive measures, with LDBC producing the most consistent improvements in strength, lifting volume, and Stroop performance, whereas HDBC produced the strongest response for Tendo-derived barbell power. Wingate anaerobic performance remained unchanged, and both doses were well tolerated. Together, these findings suggest that short-term NZBC supplementation may benefit specific resistance-exercise and cognitive measures without broadly enhancing all high-intensity performance outcomes.

The strength findings warrant attention first, as they carry the most consistent signal across conditions. Both LDBC and HDBC raised bench press and leg press 1RM above CON and exceeded PL, a pattern that held across the upper and lower body. The NZBC literature has primarily examined endurance, intermittent running, cycling performance, repeated cycling efforts, sprint-based field performance, and substrate-use outcomes, including exercise fat oxidation [1,4,5,6,7,30,31]. Resistance exercise imposes a distinct neuromuscular demand, requiring repeated high-force contractions, progressive loading, and fatigue management across sets; therefore, findings from running or cycling protocols cannot be assumed to translate directly to resistance-trained performance outcomes [23,32,33]. That both blackcurrant doses improved 1RM across upper- and lower-body lifts points to ergogenic effects in a training context that prior NZBC research has largely overlooked.

Leg press volume load followed a similar pattern. Total lifting volume was highest under LDBC, which exceeded not only CON but also PL and HDBC. HDBC also outperformed CON, though the margin was smaller. Bench press volume did not reach a significant omnibus effect after the Greenhouse–Geisser correction. However, exploratory pairwise comparisons indicated that LDBC was above CON and PL. Why the lower body responded more robustly is unclear, but one plausible explanation is that leg press exercise recruits a larger active muscle mass, thereby placing greater demands on fatigue resistance, local perfusion, and metabolic support during repeated high-force work [32,33]. It bears noting, however, that CON was conducted first in all cases, with the capsule conditions following in a randomized order; consequently, familiarization and order effects cannot be fully excluded as partial contributors to the observed improvements.

A notable finding was that PL was significantly higher than CON for leg press 1RM and leg press total lifting volume. This finding should not be interpreted as direct evidence that maltodextrin improved strength performance. The placebo capsule contained only 600 mg/day of maltodextrin, unlikely to produce a meaningful carbohydrate-mediated ergogenic effect. Instead, the PL-versus-CON difference may reflect expectancy effects related to the capsule, repeated exposure to lower-body strength and volume testing, normal day-to-day performance variability, and/or period effects. This interpretation is especially relevant because CON was completed first and was not capsule-blinded, whereas PL, LDBC, and HDBC were administered as identical capsules under blinded conditions and randomized across the remaining periods. Therefore, placebo-controlled contrasts should be emphasized when interpreting NZBC-specific efficacy. In this context, both NZBC conditions exceeded PL for leg press 1RM, and LDBC also exceeded PL for leg press total lifting volume, supporting an effect beyond the non-specific improvement observed from CON to PL.

The mechanistic basis for the resistance-exercise improvements cannot be established from this study. NZBC anthocyanins have been linked to vascular/endothelial responses and exercise-related oxidative-stress recovery [3,34], while prior NZBC studies have reported increased fat oxidation during exercise [5,31]. In repeated high-force resistance exercise, fatigue resistance, neuromuscular function, and the ability to sustain performance across sets are central to performance capacity; so, these pathways provide a biologically plausible framework for interpreting the present findings [23,32]. However, because anthocyanin metabolites, vascular function, muscle oxygenation, and substrate utilization were not assessed, it cannot be determined whether any of them actually drove the improvements observed here. Future trials in this setting would benefit from building in these endpoints from the outset.

To provide a clearer roadmap for follow-up work, future NZBC studies in resistance-trained adults should prioritize several mechanistic pathways. First, vascular and perfusion-related mechanisms should be assessed through measures such as endothelial function, nitric oxide availability, limb blood flow, and skeletal muscle oxygenation during resistance exercise. Second, redox and inflammatory responses should be evaluated using markers of oxidative stress, antioxidant capacity, and exercise-induced inflammation. Third, anthocyanin pharmacokinetics and metabolite profiling are needed to determine whether LDBC and HDBC produce different circulating metabolite patterns. Finally, intramuscular fatigue-related mechanisms, including phosphocreatine recovery, lactate accumulation, metabolite clearance, and local muscular fatigue, should be examined. These endpoints would help determine whether NZBC improves resistance-exercise performance primarily through vascular, metabolic, redox, or bioavailability-mediated pathways.

The greater leg press total lifting volume observed with LDBC compared with HDBC suggests that the ergogenic effects of NZBC may not follow a simple linear dose–response pattern. This interpretation is consistent with evidence that phytochemicals can elicit non-linear or biphasic biological responses [35]. Reduced bioavailability alone is unlikely to explain the smaller HDBC response, because Hurst et al. reported a time- and dose-dependent increase in plasma anthocyanins after ingestion of an NZBC anthocyanin-rich extract [34], and Costello et al. reported plasma uptake of selected phenolic acid metabolites following CurraNZ intake [36]. However, because plasma anthocyanins, phenolic metabolites, urinary metabolites, oxidative-stress markers, antioxidant capacity, and inflammatory responses were not measured in the present study, the underlying mechanism cannot be determined.

The LDBC formulation also contained 350 mg/day maltodextrin as the capsule filler, whereas the HDBC formulation contained the full 600 mg/day as NZBC extract. This small amount of maltodextrin is unlikely to provide a meaningful carbohydrate-mediated ergogenic effect, but a formulation or matrix effect cannot be completely excluded. It is also possible that a higher polyphenol dose does not necessarily provide additional benefit across all outcomes and could, in theory, influence redox-sensitive signaling or endogenous oxidative-stress responses. This explanation remains speculative, and the stronger LDBC response should be considered preliminary and hypothesis-generating. Future dose–response studies should use matched excipient conditions and include blood or urinary markers of anthocyanin and phenolic acid metabolites, oxidative stress, antioxidant capacity, and inflammatory responses.

Regarding Tendo-derived power, HDBC exceeded PL for both peak and mean power during bench press work sets and surpassed LDBC for mean power. These differences are practically relevant because power output reflects the neuromuscular capacity to generate force rapidly and maintain movement velocity, which is distinct from total lifting volume [37]. That HDBC significantly outperformed PL in a double-blinded comparison is a meaningful finding. Neither Tendo outcome, however, reached significance versus CON for HDBC, and the power findings are therefore best interpreted as preliminary evidence of a high-dose advantage rather than a definitive conclusion.

The lack of change in Wingate performance is an important finding and suggests that the ergogenic effects observed for selected resistance outcomes did not generalize to all high-intensity tasks. This likely reflects task specificity. Bench press and leg press outcomes were familiar resistance-training tasks involving external loading, movement-specific coordination, repeated high-force contractions, local fatigue resistance, and recovery between sets. In contrast, the Wingate test is a continuous 30 s all-out cycling sprint with distinct mechanical, neuromuscular, and fatigue demands. Although well validated as an anaerobic test [11,12,13], it does not replicate the movement pattern, loading strategy, or intermittent nature of resistance exercise. Similar null findings have been reported after acute and 7-day supplementation with blackcurrant extract in trained cyclists [8] and after several days of NZBC intake during sustained isometric work [9].

Differences in muscle mass involvement and energy system demands may also explain the divergent findings. Leg press total lifting volume involves repeated contractions of a large lower-body muscle mass under external load, with rest intervals between sets, and may be influenced by local perfusion, vascular responsiveness, phosphocreatine recovery, metabolite clearance, and fatigue tolerance. By contrast, the Wingate test compresses performance into a single maximal 30 s cycling bout and depends heavily on immediate phosphagen availability, rapid glycolytic energy production, cycling-specific power output, and tolerance to severe acidosis [38]. Therefore, mechanisms potentially influenced by NZBC may be more relevant to repeated-set resistance exercise than to a single short-duration cycling sprint.

The selection of the 30 s Wingate test rather than an aerobic capacity test should be interpreted in relation to the study aim. This trial focused on resistance-trained adults and high-intensity outcomes relevant to strength, lifting volume, barbell power, and anaerobic performance. Therefore, the Wingate test was chosen as a validated anaerobic assessment aligned with this study’s performance focus. However, because cardiopulmonary fitness is often associated with cognitive function, the absence of VO_2_max or another aerobic fitness measure limits the interpretation of whether baseline aerobic capacity contributed to Stroop performance.

As regards cognition, Stroop scores improved across all test sections under both blackcurrant conditions, with LDBC yielding the most consistent gains. That PL outperformed CON is worth noting, as it suggests repeated task exposure contributed to score gains. Even so, LDBC exceeded PL on Color, Color-Word, and total score, making these improvements less likely to be explained by practice alone. Comparable findings appear elsewhere in the blackcurrant literature: Watson et al. found that acute blackcurrant extract supplementation both modulated cognitive performance and reduced MAO-B activity in healthy young adults, while Gibson et al. documented better mental performance in rugby league players after one week on a blackcurrant-based nootropic formulation. Our data extend these findings to resistance-trained adults undergoing short-term NZBC supplementation, with cognitive improvements that remained significant after accounting for placebo and practice contributions.

This pattern also highlights why supplement-versus-CON contrasts were interpreted cautiously. The PL advantage over CON on the Stroop Word section indicates that repeated task exposure, expectancy, and/or order effects likely contributed to some cognitive gains. The data do not support an inherent cognitive-enhancing effect of maltodextrin, given the very small placebo dose (600 mg/day). Accordingly, NZBC-specific cognitive interpretation was based primarily on outcomes that exceeded PL; where the blackcurrant conditions did not differ from PL, the findings were interpreted more cautiously.

The neurobiological mechanisms underlying the Stroop improvements cannot be determined from this dataset. The most plausible neurochemical candidate is MAO-B inhibition, which prior blackcurrant extract studies have linked to cognitive effects in healthy adults [17]. Whether anthocyanin-related vascular or oxidative-stress pathways also contributed is uncertain. These mechanisms are biologically plausible based on prior NZBC work [3,34], but the present study did not include cerebrovascular, oxidative stress, or neurochemical measures. Determining which, if any, of these mechanisms was operative would require targeted mechanistic outcomes not included in this study.

Readiness-to-perform scores were comparable across all conditions at the start of each session, indicating that participants did not arrive in a meaningfully different subjective state across supplemented and non-supplemented visits. Post-exercise RPE was elevated following bench press and leg press in the blackcurrant conditions relative to CON. This finding should not be interpreted as evidence of reduced tolerability. Participants lifted considerably more weight under the blackcurrant conditions, particularly in the leg press. Hence, the higher RPE values are more readily attributed to a greater physical workload than to any perceptual effect of the supplement itself [39,40].

Resting and post-exercise HR and BP did not differ in any meaningful way across conditions, and nothing on the side-effects questionnaire pointed to treatment-related concerns. These findings support the short-term tolerability of both NZBC doses in young resistance-trained adults. They are consistent with prior NZBC supplementation studies in active or trained participants that reported no major adverse physiological or cardiovascular concerns [8,9,30].

Practitioners working in resistance training settings may find the selective performance improvements practically relevant, though the findings warrant careful interpretation. Not all outcomes responded favorably; the Wingate was entirely unaffected by either dose, and the dose-by-outcome dissociation complicates straightforward supplementation recommendations. Some portion of the observed Stroop gains also likely reflects repeated task exposure and expectancy rather than a direct pharmacological action of NZBC.

The within-subject crossover structure was the most consequential design feature of this study. With all participants completing all four conditions, the design reduced between-participant variability in treatment comparisons, a meaningful advantage at *n* = 20, where individual differences could easily obscure modest supplement effects. The outcome battery covered maximal strength, total lifting volume, Tendo-derived power, Wingate anaerobic capacity, Stroop cognitive performance, perceptual and hemodynamic responses, and adverse events, providing a broad characterization of where NZBC does and does not appear to matter in this population. Recruiting both males and females added ecological validity, and testing two doses rather than one revealed the dose-by-outcome dissociation, which emerged as one of the more informative findings in the dataset.

Several limitations constrain the interpretation of these findings. The most structurally consequential is the non-randomized CON condition. Conducting CON in period 1 for all participants provided a stable capsule-free baseline. Still, it introduced the possibility that learning, familiarization, or short-term training adaptations contributed to performance improvements in subsequent supplemented conditions. These effects cannot be cleanly partitioned from supplementation-related changes. Practice effects on the Stroop were evident under PL, and the breadth of the outcome battery increased the number of statistical comparisons. Therefore, pairwise findings for some outcomes, particularly bench press lifting volume, should be regarded as exploratory rather than confirmatory [41]. The absence of mechanistic endpoints leaves the biological basis of the observed performance and cognitive changes unresolved. Dietary records were self-reported, introducing the usual problems of recall error and portion estimation. The sample was also drawn exclusively from young resistance-trained adults; so, it remains an open question whether these findings translate to older individuals, clinical populations, or longer supplementation periods.

Menstrual-cycle phase and hormonal contraceptive status were not prospectively controlled, which may have contributed to variability in exercise, perceptual, or hemodynamic responses among female participants. Future studies should consider standardizing testing by menstrual-cycle phase and/or stratifying analyses by hormonal contraceptive status.

The sample was not balanced by sex, and this study was not powered to detect sex-specific treatment responses; therefore, sex-specific treatment effects were not analyzed. Future studies with larger, prospectively balanced samples should examine whether biological sex modifies the response to NZBC supplementation.

Since CON was completed first and involved no capsule administration, comparisons between CON and the capsule conditions may include non-specific effects related to expectancy, repeated testing, order, and normal performance variability. This limitation applies to both physical and cognitive outcomes. Therefore, placebo-controlled comparisons are the most appropriate basis for interpreting NZBC-specific efficacy, and outcomes that did not exceed PL should not be interpreted as clear supplement-specific effects.

These mechanistic limitations also affect the interpretation of the apparent non-linear, outcome-specific dose–response pattern. Therefore, the possibility that LDBC produced a more favorable physiological or metabolite profile than HDBC remains plausible but unconfirmed.

Aerobic capacity was not assessed. Although the Wingate test was selected to align with this study’s focus on strength and high-intensity performance, the absence of VO_2_max or another cardiopulmonary fitness measure limits the interpretation of potential relationships between aerobic fitness and Stroop performance. Future NZBC studies that include cognitive outcomes should consider assessing aerobic fitness alongside anaerobic and resistance performance measures.

On the methodological side, the most pressing fix for future work is straightforward: the CON condition should be randomized alongside the capsule conditions rather than fixed at period 1, thereby allowing supplementation effects to be more cleanly separated from learning and order contributions. Sample sizes will need to be larger to reliably detect sex-specific patterns. Targeted mechanistic endpoints, including muscle oxygenation, vascular function, oxidative stress markers, anthocyanin metabolite concentrations, and cerebral perfusion, are also needed if future work is to explain why certain outcomes responded, and others did not. Dose–response investigations across a wider dose range are also warranted, given the outcome-specific dissociation between the 250 and 600 mg·day^−1^ conditions observed here.

## 5. Conclusions

Short-term NZBC supplementation improved selected resistance-exercise and cognitive outcomes in resistance-trained adults, while Wingate anaerobic performance was unaffected by either dose. The strongest evidence for NZBC-specific efficacy was observed for outcomes that exceeded both CON and PL, particularly maximal strength and the LDBC response for lower-body lifting volume. The significant PL response relative to CON indicates that non-specific capsule-related expectancy, repeated testing, period, or normal performance-variability effects may have contributed to some of the lower-body improvements; therefore, placebo-controlled contrasts should be emphasized when interpreting the findings. The pattern of effects was task-specific and dose-dependent, with LDBC producing more consistent benefits for strength and cognitive outcomes, and HDBC showing stronger effects on barbell power. Neither dose raised any tolerability concerns over the seven days. Independent replication in larger samples, with randomized capsule-free control conditions and mechanistic endpoints built into the design from the outset, is the logical next step before the observed effects can be considered established.

## Figures and Tables

**Table 1:** Baseline characteristics of participants.

Variable	Male (*n* = 7) Mean ± SD	Female (*n* = 13) Mean ± SD	Total (*n* = 20) Mean ± SE
Age (y)	21.6 ± 0.79	21.5 ± 0.52	21.6 ± 0.60
Height (cm)	174.6 ± 6.02	164.5 ± 5.86	168.0 ± 7.59
Weight (kg)	83.7 ± 9.03	68.7 ± 17.10	73.9 ± 16.3
Body fat (%)	18.0 ± 6.66	29.1 ± 8.98	25.2 ± 9.73
Bench press 1RM (kg)	102.4 ± 34.2	36.6 ± 11.4	59.6 ± 38.5
Leg press 1RM (kg)	362.0 ± 65.3	198.5 ± 57.1	255.7 ± 99.0

Data are presented descriptively as mean ± SD for males and females and mean ± SE for the total sample. 1RM = one-repetition maximum.

**Table 2:** Dietary analysis.

Variable	Treatment	Total (*n* = 20) Mean ± SE	*p*-Value
Energy intake	CON	1969.3 ± 677.8	0.07
	PL	1931.3 ± 674.2	
	LDBC	1883.4 ± 577.5	
	HDBC	1804.7 ± 670.8	
Protein	CON	94.3 ± 41.9	0.75
	PL	91.6 ± 46.9	
	LDBC	92.8 ± 40.7	
	HDBC	90.2 ± 44.6	
Carbohydrate	CON	230.8 ± 104.3	0.06
	PL	204.5 ± 68.8	
	LDBC	203.0 ± 48.4	
	HDBC	185.5 ± 71.8	
Fat	CON	76.6 ± 28.6	0.54
	PL	76.5 ± 30.1	
	LDBC	79.3 ± 26.5	
	HDBC	73.6 ± 28.4	

Values are presented as mean ± SE from 3-day dietary records for each condition. Energy intake is reported in kcal/day, and macronutrients are reported in g/day. *p*-values reflect the main effect of condition across CON, PL, LDBC, and HDBC.

**Table 3:** Resistance Exercise Performance.

Variable	Treatment	Male (*n* = 7) Mean ± SD	Female (*n* = 13) Mean ± SD	Total (*n* = 20) Mean ± SE	*p*-Value
Bench press 1RM (kg)	CON	102.7 ± 34.2	37.3 ± 11.8	70.0 ± 5.16 ^c,d^	0.003 ***
	PL	103.7 ± 32.9	37.3 ± 11.3	70.5 ± 4.95 ^c,d^	
	LDBC	106.9 ± 30.8	39.8 ± 11.6	73.3 ± 4.73 ^a,b^	
	HDBC	105.3 ± 39.4	39.4 ± 10.7	72.4 ± 4.80 ^a,b^	
Leg press 1RM (kg)	CON	367.5 ± 75.8	206.9 ± 57.1	287.2 ± 16.4 ^c,d^	<0.001 ***
	PL	381.6 ± 84.4	210.1 ± 64.9	296.0 ± 16.8 ^a,c,d^	
	LDBC	418.5 ± 86.5	230.0 ± 59.7	324.4 ± 16.3 ^a,b^	
	HDBC	405.7 ± 92.1	218.8 ± 62.2	313.0 ± 17.2 ^a,b^	
Bench press total lifting volume (kg)	CON	2100.8 ± 898.5	753.3 ± 254.7	1427.1 ± 131.0	0.102
	PL	2134.7 ± 814.2	784.6 ± 185.3	1459.6 ± 115.7	
	LDBC	2181.5 ± 781.9	896.2 ± 249.2	1538.9 ± 116.1	
	HDBC	2090.8 ± 696.4	851.8 ± 328.0	1471.3 ± 113.2	
Leg press total lifting volume (kg)	CON	9288.5 ± 2279.0	4504.2 ± 1583.8	6896.4 ± 432.4 ^b,c,d^	<0.001 ***
	PL	9685.6 ± 2699.0	5147.3 ± 2112.1	7416.5 ± 544.8 ^a,c^	
	LDBC	12,484.0 ± 4266.8	6562.8 ± 2023.4	9523.4 ± 695.2 ^a,b,d^	
	HDBC	10,498.5 ± 3705.1	5343.4 ± 1828.8	7920.9 ± 611.5 ^a,c^	
Tendo peak power (W)	CON	501.8 ± 177.3	161.7 ± 56.4	331.8 ± 26.3	0.017 *
	PL	446.4 ± 168.9	169.2 ± 49.3	307.8 ± 24.7 ^d^	
	LDBC	469.6 ± 166.4	192.0 ± 66.8	330.8 ± 25.9	
	HDBC	543.8 ± 169.4	230.9 ± 104.8	387.4 ± 30.4 ^b^	
Tendo mean power (W)	CON	346.7 ± 113.9	112.8 ± 39.3	229.8 ± 17.1	0.038 *
	PL	305.1 ± 86.9	120.3 ± 32.8	212.7 ± 13.3 ^d^	
	LDBC	301.0 ± 83.5	123.2 ± 31.5	212.1 ± 12.8 ^d^	
	HDBC	359.8 ± 108.1	158.1 ± 71.8	258.9 ± 20.1 ^b,c^	

Values are presented descriptively as mean ± SD for males and females and as model-based estimated marginal means ± SE for the total sample. Male and female values are presented descriptively only and were not used for sex-specific inferential comparisons. p-values represent the main effect of treatment across CON, PL, LDBC, and HDBC. Superscript letters in the total column indicate significant post hoc pairwise differences among treatment conditions: ^a^ different from CON, ^b^ different from PL, ^c^ different from LDBC, and ^d^ different from HDBC (*p* < 0.05). For outcomes with a non-significant overall treatment effect, pairwise findings should be interpreted as exploratory. Asterisks indicate statistical significance for the overall treatment effect: * *p* < 0.05 and *** *p* < 0.001. CON = control; PL = placebo; LDBC = low-dose blackcurrant; HDBC = high-dose blackcurrant.

**Table 4:** Wingate anaerobic performance.

Variable	Treatment	Male (*n* = 7) Mean ± SD	Female (*n* = 13) Mean ± SD	Total (*n* = 20) Mean ± SE	*p*-Value
Peak Power (W)	CON	866.3 ± 225.9	560.3 ± 135.3	718.9 ± 40.0	0.102
	PL	868.4 ± 186.1	550.2 ± 134.8	709.4 ± 36.1	
	LDBC	782.4 ± 145.1	549.1 ± 119.2	665.8 ± 30.1	
	HDBC	759.1 ± 166.7	572.5 ± 123.4	665.8 ± 32.6	
Mean Power (W)	CON	566.2 ± 137.3	392.1 ± 78.5	481.0 ± 23.9	0.611
	PL	567.4 ± 117.6	380.6 ± 83.7	477.7 ± 22.5	
	LDBC	561.2 ± 91.1	389.3 ± 82.6	477.2 ± 20.0	
	HDBC	545.4 ± 97.0	391.9 ± 75.2	468.6 ± 19.4	
Total Work (J)	CON	14,785.2 ± 5300.4	11,537.1 ± 2718.2	13,161.2 ± 886.1	0.784
	PL	15,286.1 ± 5018.7	11,368.2 ± 2547.6	13,327.2 ± 836.1	
	LDBC	15,256.7 ± 4057.0	11,633.7 ± 2442.9	13,445.2 ± 721.1	
	HDBC	15,010.7 ± 4014.6	11,504.5 ± 2394.5	13,257.6 ± 710.7	

Values are presented descriptively as mean ± SD for males and females and as model-based estimated marginal means ± SE for the total sample. Male and female values are presented descriptively only and were not used for sex-specific inferential comparisons. *p*-values represent the main effect of treatment across CON, PL, LDBC, and HDBC. CON = control; PL = placebo; LDBC = low-dose blackcurrant; HDBC = high-dose blackcurrant.

**Table 5:** Cognitive Function.

Variable	Treatment	Male (*n* = 7) Mean ± SD	Female (*n* = 13) Mean ± SD	Total (*n* = 20) Mean ± SE	*p*-Value
Word (counts)	CON	106.2 ± 10.9	106.0 ± 15.7	106.1 ± 3.35 ^b,c,d^	<0.001 ***
	PL	120.5 ± 7.10	118.2 ± 14.9	119.4 ± 3.02 ^a^	
	LDBC	120.3 ± 8.60	126.1 ± 16.3	123.1 ± 3.33 ^a,d^	
	HDBC	116.7 ± 3.03	120.7 ± 16.7	118.7 ± 3.22 ^a,c^	
Color (counts)	CON	81.0 ± 11.5	78.6 ± 10.4	79.8 ± 2.54 ^b,c,d^	<0.001 ***
	PL	86.7 ± 11.4	82.1 ± 12.1	84.4 ± 2.78 ^a,c^	
	LDBC	88.8 ± 11.5	90.2 ± 13.7	89.5 ± 3.70 ^a,b,d^	
	HDBC	85.1 ± 13.0	84.6 ± 15.7	84.9 ± 3.49 ^a,c^	
Color–Word (counts)	CON	99.2 ± 16.9	97.8 ± 16.4	98.5 ± 3.88 ^c,d^	0.002 **
	PL	101.8 ± 11.5	106.7 ± 17.2	104.3 ± 3.64 ^c,d^	
	LDBC	113.1 ± 14.6	116.1 ± 16.2	114.6 ± 3.69 ^a,b^	
	HDBC	110.8 ± 8.87	110.2 ± 20.1	110.5 ± 4.04 ^a,b^	
Total Stroop (counts)	CON	286.5 ± 18.6	282.4 ± 37.9	284.5 ± 7.68 ^b,c,d^	<0.001 ***
	PL	309.1 ± 27.8	307.1 ± 40.2	308.1 ± 8.58 ^a,c^	
	LDBC	322.2 ± 31.8	332.4 ± 40.4	327.3 ± 8.85 ^a,b,d^	
	HDBC	312.7 ± 21.2	315.6 ± 48.3	314.2 ± 9.68 ^a,c^	

Values are presented descriptively as mean ± SD for males and females and as model-based estimated marginal means ± SE for the total sample. Male and female values are presented descriptively only and were not used for sex-specific inferential comparisons. Exact *p*-values represent the main effect of treatment across CON, PL, LDBC, and HDBC. Superscript letters in the total column indicate significant post hoc pairwise differences among treatment conditions: ^a^ different from CON, ^b^ different from PL, ^c^ different from LDBC, and ^d^ different from HDBC (*p* < 0.05). Asterisks indicate statistical significance for the overall treatment effect: ** *p* < 0.01 and *** *p* < 0.001. CON = control; PL = placebo; LDBC = low-dose blackcurrant; HDBC = high-dose blackcurrant.

## Data Availability

The de-identified data supporting the findings of this study are not publicly available but may be made available from the corresponding author upon reasonable request, subject to institutional and ethical approval requirements.

## Abbreviations

The following abbreviations are used in this manuscript:

1RM	One-repetition maximum
ANOVA	Analysis of variance
BP	Blood pressure
CI	Confidence interval
CON	No-capsule control
DBP	Diastolic blood pressure
DXA	Dual-energy X-ray absorptiometry
ES	Effect size
HDBC	High-dose blackcurrant
HR	Heart rate
LDBC	Low-dose blackcurrant
NZBC	New Zealand blackcurrant
PL	Placebo
RPE	Rating of perceived exertion
SBP	Systolic blood pressure
SD	Standard deviation
SE	Standard error
TRT	Treatment
VAS	Visual analog scale
